# Sex Differences in Ischemic Stroke Within the Younger Age Group: A Register-Based Study

**DOI:** 10.3389/fneur.2022.793181

**Published:** 2022-02-14

**Authors:** Kristina Norman, Marie Eriksson, Mia von Euler

**Affiliations:** ^1^School of Medicine, Örebro University, Örebro, Sweden; ^2^Department of Statistics, USBE, Umeå University, Umeå, Sweden; ^3^Department of Neurology and Rehabilitation, School of Medicine, Örebro University, Örebro, Sweden

**Keywords:** ischemic stroke, register, sex differences, younger population, cerebrovascular disease

## Abstract

**Background:**

Stroke incidence is decreasing in most developing countries. However, worrisome trends of an increase in the younger population have been described.

**Aim:**

To investigate sex differences and longitudinal changes in ischemic stroke regarding incidence, cardiovascular risk factors, and outcome, in the young.

**Methods:**

This is an observational study based on the data from the Swedish national stroke registry, Riksstroke. Patients, 18–54 years of age, having ischemic stroke between 2005 and 2018 were included, resulting in a study population of 16,210 patients.

**Results:**

The incidence was higher in men than in women (30.6 vs. 19.1 per 100,000, *P* < 0.001). After an initial increase, the incidence stabilized and then decreased, resulting in a similar level in 2018 as in 2005. Atrial fibrillation, diabetes, and usage of anti-hypertensives at stroke onset were more common among men and did not change over time. Smoking was common and slightly more so in women, but with a reduced prevalence in both men and women during the study period. Dependency in Activities of Daily Living (ADL) and case fatality showed no clear trends or sex differences.

**Conclusions:**

The results show that there are sex differences in ischemic stroke in the younger age group regarding incidence and vascular risk factors, particularly smoking. Temporal trends in stroke incidence are difficult to interpret as fluctuations are substantial, largely due to stroke being quite uncommon in the younger population.

## Introduction

Age and sex are important non-modifiable risk factors for stroke. Women are older at the time of stroke onset but have a longer life expectancy (1). Thus, the absolute number of strokes is lower for women before the age of 75 years but more than twice as high as in men after the age of 75 (2). Protective effects of physiological estrogen until menopause (3), and more men having stroke risk factors such as smoking and untreated vascular risk factors have been suggested as explanations (4, 5). The incidence is higher among men than women below the age of 60. In a study of 411 patients between 18 and 50 years of age from China, 67% were male and 88% of the male patients had at least one risk factor, compared to 54% of the women (4). Some of the risk factors observed more often in men were hypertension, diabetes mellitus, and smoking (4).

A trend of increasing incidence of stroke in the younger age group in Sweden has previously been described. One such report by Rosengren et al. in 2013 based on Swedish Hospital Discharge and Cause of Death (CDR) registries researched cases of ischemic stroke from 1987 to 2010. The study showed that there was a continuous increase in the incidence of ischemic stroke for both men and women in the age group 18 to 44 years, although slightly larger among women (+1.3 in men, +1.6 in women) (6). Factors that were hypothesized to contribute were increasing obesity rates but also increased detection of smaller strokes. Nevertheless, the report describes this worrisome trend and its possibility to be carried forward as this younger population grows older. An increase in incidence in a slightly older age group, 35–64 years, in the Netherlands has also been found in the years 1997 to 2005 (7). As the number of strokes in younger age groups is small, a lesser change in the number of cases results in a large change in proportions. Both the number of strokes and the case fatality (CF) have continued to decrease in men and women in the older age groups and thus, it is interesting to follow the incidence over time in the younger age groups. We aimed to investigate sex differences in ischemic stroke regarding incidence, risk factors, and case fatality in the younger populations.

## Materials and Methods

This is an observational register-based study based on prospectively collected data from the Swedish national quality registry for stroke, Riksstroke. The registry's coverage of stroke incidence is high, calculated to 89% when compared to the patient registry (PAR) (8). The study population consisted of men and women between the ages of 18 and 54 and is a subset of a data set that has been published previously (9). Both first-ever and recurrent ischemic strokes (ICD-10 code I63) were included.

The risk factors diabetes, high blood pressure (assessed as using anti-hypertensive medication), previous history of stroke (yes or no), smoking, and atrial fibrillation are recorded in Riksstroke. Smoking is defined as the consumption of one cigarette or more per day, or if the patient had quit during the 3 months leading up to the stroke attack. The date of death was retrieved from the Cause of Death Register (managed by the National Board of Health and Welfare), giving statistics of 90 days-CF. Dependency in ADL was based on the patient's reported outcome (mobility, toileting, and dressing) 3 months after stroke.

### Statistical Analysis

Age (years) was non-normally distributed and described with medians and quartiles (Q1 and Q3). Binary variables were presented by proportions with approximately 95% CIs. Group comparisons were made with the Mann-Whitney test for age and Pearson's chi-squared test for categorical variables.

The stroke incidence was analyzed by Poisson regression, with an offset equal to the natural logarithm of the size of the general population (using population statistics from Statistics Sweden, Central Bureau of Statistics). The model included the independent variables year, sex, and sex-by-year interaction. Risk factors and outcomes were analyzed by binary logistic regression. The models included the independent variables sex, year of stroke, age, and age^2^. In addition, the sex-by-year interaction was included to test if the time trend differed between men and women.

A *p* < 0.05 was considered statistically significant. Statistical analysis was performed using IBM SPSS® statistics version 26.0 for Windows (IBM Corp., Armonk, NY, USA), and SAS software, Version 9.4 of the SAS System for Windows (SAS Institute Inc., Cary, NC, USA).

### Ethical Considerations

This study was approved by the Swedish Ethical Review Authority (reference no. 2018-02777). In accordance with the Personal Data Act (Swedish law No. SFS 1998:204), no informed consent is needed to collect data from medical charts and inpatient records for quality registers but there is information and an opt-out possibility.

## Results

**Figure 1:**
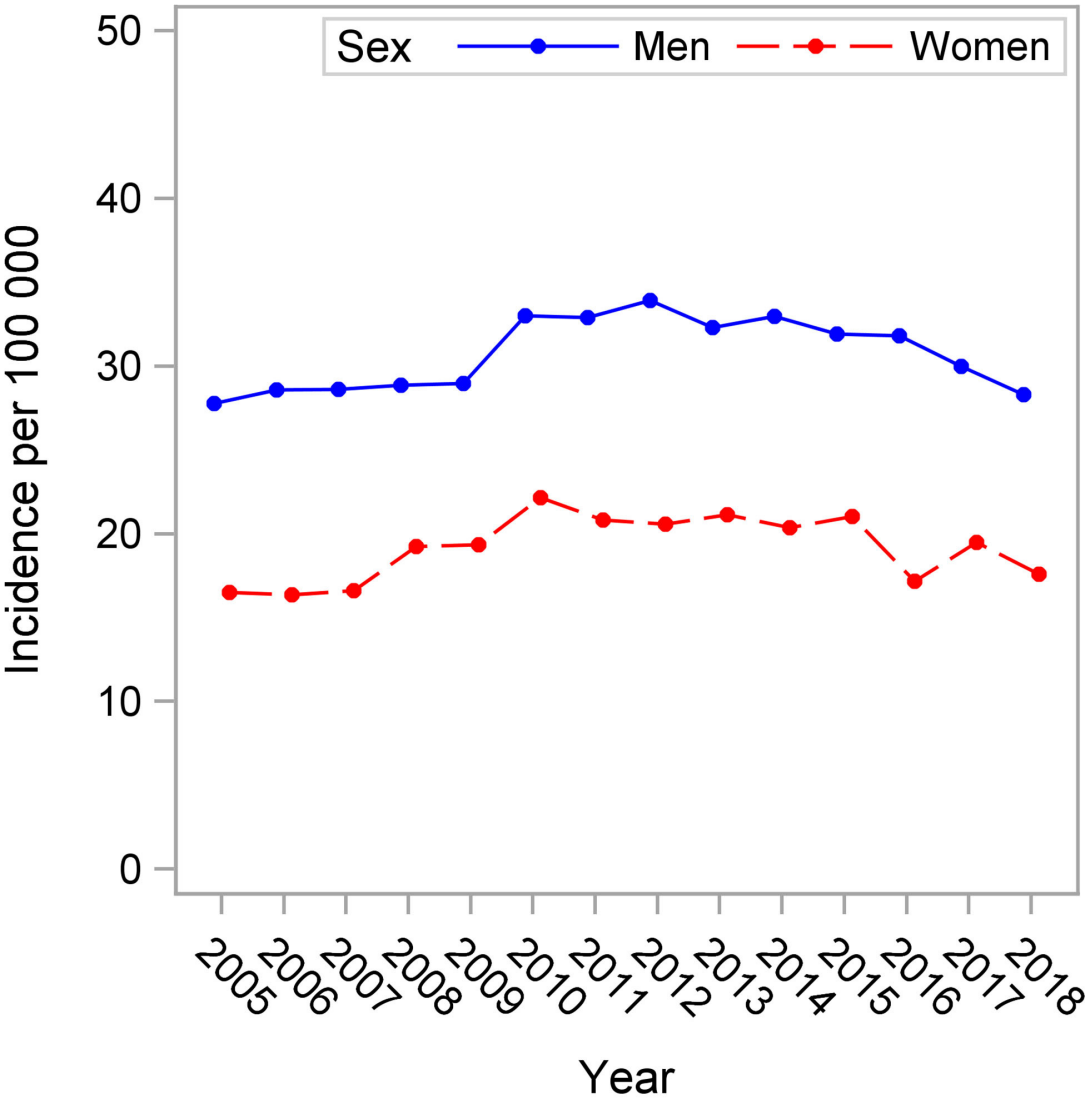
Estimated stroke incidence per 100,000 in 18–54 years old men and women, 2005–2018.

In all, 16,210 persons were included (6,073 women, 10,137 men). Throughout 2005–2018, the estimated stroke incidence was higher in men than in women (30.6 per 100,000 in men and 19.1 per 100,000 in women, *P* < 0.001, Figure 1). The incidence changed significantly over the years (*P* < 0.001) similarly in men and women (P for sex-by-year interaction = 0.278). There was a slight increase between 2005 and 2010 the trend levels off after this, and in 2018 the incidence rates for both men and women were almost at the same level as in 2005.

**Figure 2:**
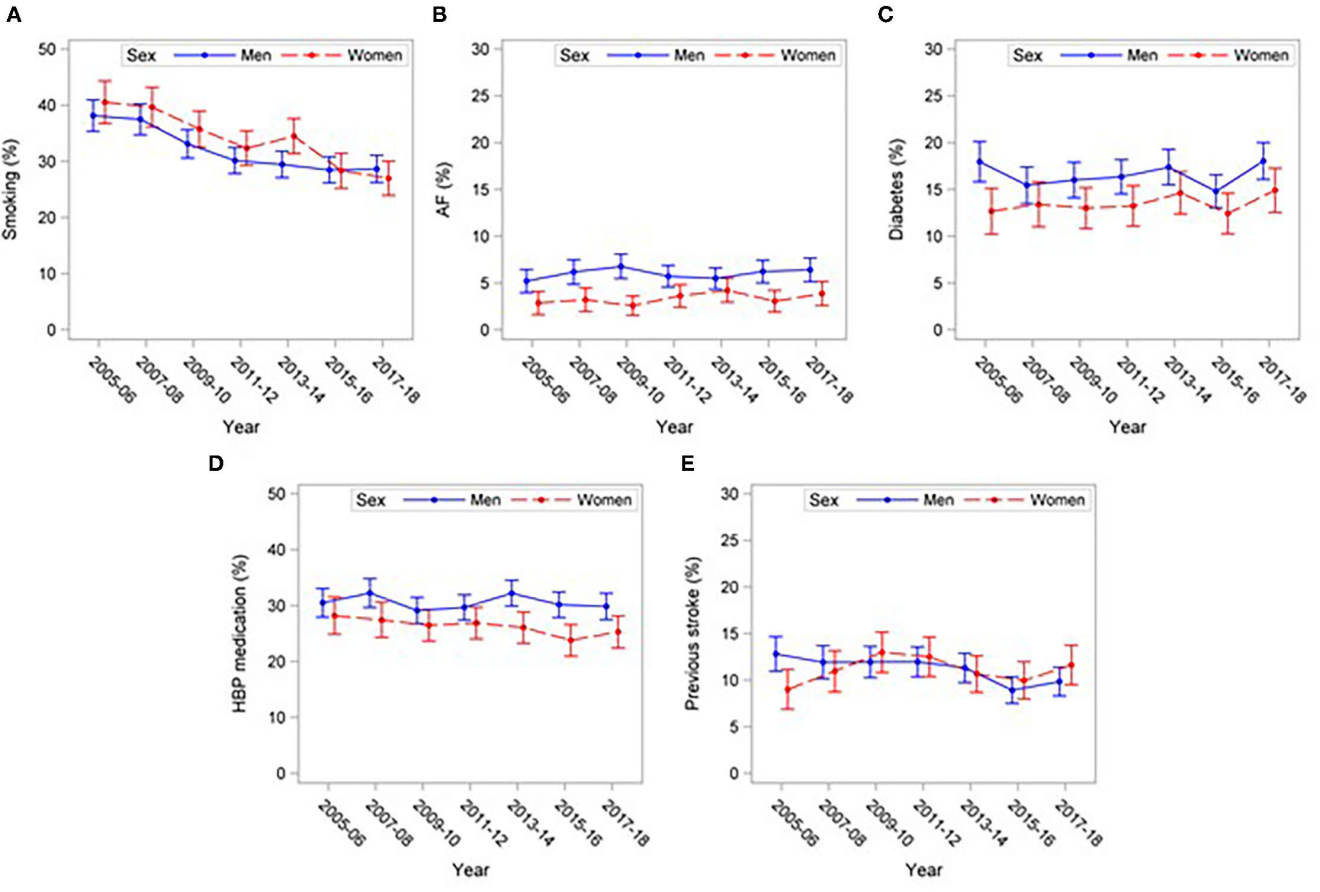
Prevalence of cardiovascular risk factors (%) with 95% CIs in 18–54-year-old patients with stroke 2005–2018. **(A)** Smoking, **(B)** Atrial fibrillation (AF), **(C)** Diabetes, **(D)** High Blood Pressure (HBP) medication, **(E)** Previous stroke.

Smoking decreased during the study period (*P* < 0.001), from 38.3% in 2005 to 25.8% in 2018 (Figure 2). This was the only studied risk factor that changed significantly during the study (Figure 2). Atrial fibrillation, diabetes, and use of antihypertensive medication were all significantly more common among men than women (Table 1), and as for previous stroke, they did not change over time (Figure 2). The prevalence of all risk factors increased with age for both men and women (Supplementary Table 1). After adjustment for age, smoking was significantly more common in women than in men (*P* = 0.018).

**Table 1:** Baseline characteristics of the cohort.

	**All**	**Men**	**Women**	***P*** **-value**
**Demographics, Frequency, ***N*** (%)**	16,210 (100)	10,137 (62.5)	6,073 (37.5)	
Age, median (Q25 and Q75), years	49 (43 and 52)	49 (44 and 52)	48 (42 and 52)	<0.001*
**Risk factors**				
Atrial fibrillation, *N* (%) [95% CI]	810 (5.0)	606 (6.0) [5.6–6.5]	204 (3.4) [2.9–3.8]	<0.001*
Diabetes *N* (%) [95% CI]	2,487 (15.4)	1,670 (16.6) [15.8–17.3]	817 (13.5) [12.7–14.4]	<0.001*
Hypertensive blood pressure (HBP) medication, *N* (%) [95% CI]	4,656 (28.9)	3,071 (30.5) [29.6–31.4]	1,585 (26.2) [25.1–27.4]	<0.001*
Smoking^#^, *N* (%) [95% CI]	4,924 (30.4)	3,012 (29.7) [28.8–30.1]	1,912 (31.5) [30.3–32.7]	0.060
Previous stroke, *N* (%) [95% CI]	1,805 (11.2)	1,129 (11.2) [10.6–11.8]	676 (11.2) [10.4–12.0]	0.986
None of the above, *N* (%) [95% CI]	6,039 (37.3)	3,672 (36.2) [35.3–37.2]	2,367 (39.0) [37.8–40.2]	0.007*

* *Statistically significant p-value*

# *in 6.9% of cases (6.8% and 7.0% in men and women, respectively) data on smoking were missing*.

*N, number of subjects or events; CI, confidence interval*.

*All percentages (%) refer to the proportion of that column, except the top row which refers to the proportion of total subjects*.

**Figure 3:**
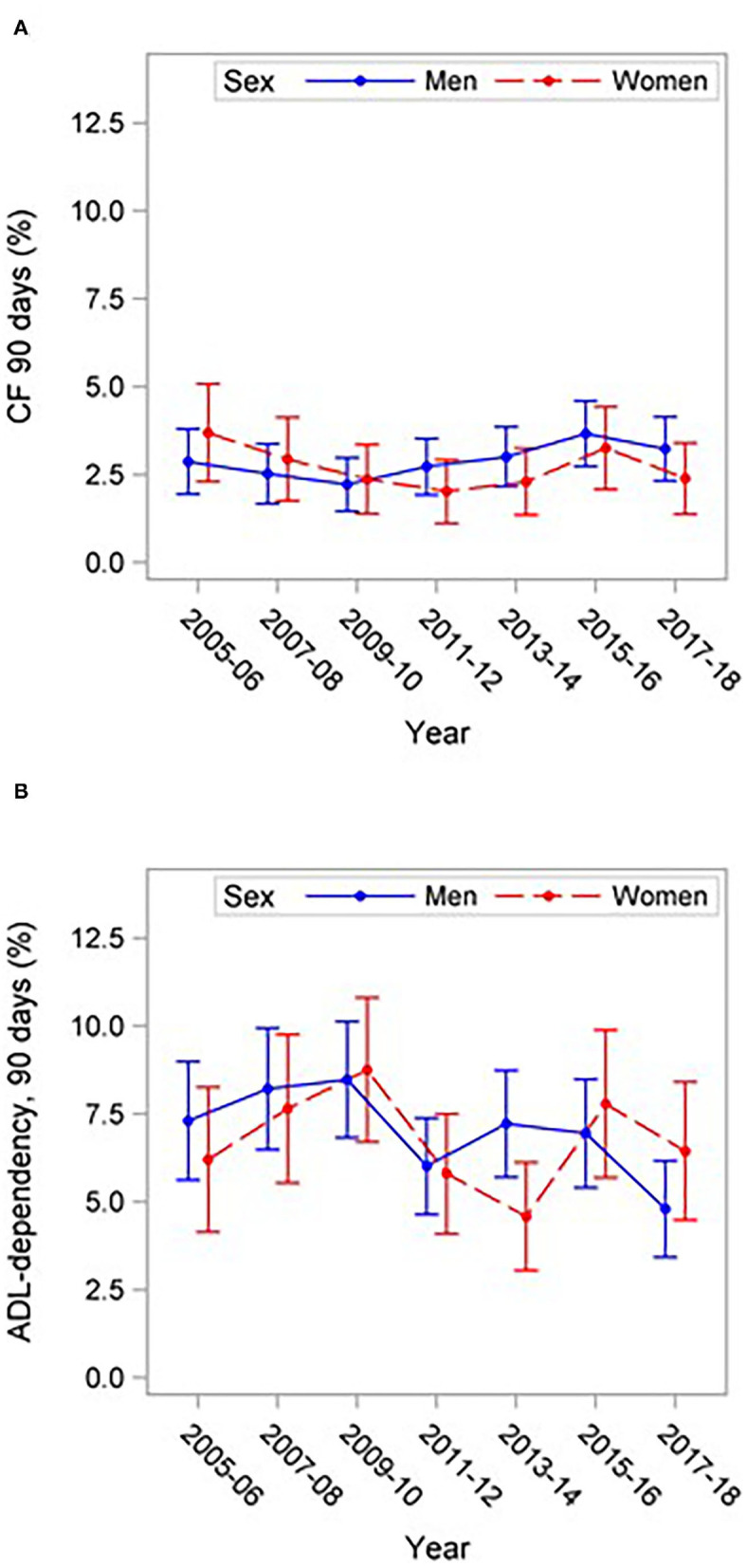
Outcome 90 days after stroke (%) with 95% CIs in 18–54-year-old patients with stroke 2005–2018. **(A)** 90 days case fatality, **(B)** ADL-dependency in patients who were independent before the stroke.

No clear sex differences could be seen for ADL-dependency at 3 months after stroke (Figure 3). Similarly, there was no consistent difference in case fatality between the sexes as some years, men have higher case fatality and other years, women (Figure 3). Overall, ADL-dependency in patients who were independent before a stroke and case fatality at 3 months after stroke did not differ significantly between men and women and did not change significantly during 2005–2018.

## Discussion

In contrast to the declining stroke incidence among the entire stroke population seen in Sweden and most other affluent countries (6, 10, 11), we observed an initial increase in incidence that was later reversed, resulting in similar levels in 2005 and 2018 in the younger population (18–54 years) in Sweden. Men had higher stroke incidence and more often atrial fibrillation, diabetes, and hypertension than women at stroke onset. Smoke prevalence in patients with stroke decreased substantially over the study period. The outcome was similar in men and women and did not change significantly over time.

The incidence was a bit lower than what has been described from an American cohort where the stroke incidence (both ischemic and hemorrhagic) was 28/100, 000 in adults 20–44 years of age (12). A recent analysis of Riksstroke data for all adults from our group shows a declining incidence of 24% in men and 31% in women during the last 15 years (9). Stroke etiology in younger people is less due to the traditional risk factors and improvements in control of these may thus have a greater impact in the older age groups. An important risk factor for ischemic stroke where treatment has improved immensely over the last decade is atrial fibrillation, which increases in prevalence with age (13, 14). In our cohort, atrial fibrillation was approximately three times as common in 42–54-year-olds compared to 18–29-year-olds. Swedish data estimate that around 10% of the reduction in ischemic stroke incidence can be related to increased oral anticoagulants treatment, especially non-vitamin K antagonist oral anticoagulants (11). Assumably, as atrial fibrillation is less common in younger people, this reduction is not as apparent in the younger age group.

Smoking became less common in the studied stroke population over the studied period. At the time of the study start, around 40% of the patient were smoking compared to 30% at the study end. Compared to the overall Swedish population, this is still a high number. From 2006 to 2020, daily smoking in Sweden has decreased from 14 to 7%, and in the youngest group (16–29 years), it has more than halved, from 10 to 4% (15). In many countries, smoking is a risk factor more commonly found in men and it is hypothesized to contribute to the incidence patterns with more men affected by stroke in the younger age group (1, 2, 4). In the present study, we only found a small difference with slightly more women than men smoking. The large difference of smokers in our cohort compared with the general population could indicate that smoking is an important risk factor in this age group. A recent meta-analysis estimated the odds ratio for stroke in smokers as compared with non-smokers to be 1.61 (95% CI 1.34–1.93, *P* < 0.001) (16). However, if it is smoking by itself or a combination of smoking and other risk factors such as low socioeconomic status and stress co-variating with smoking, cannot be deduced from our data as we lack information on several risk factors (17). Smoking being much more common in younger stroke patients compared to the entire stroke population was also shown in a study from the Dijon stroke registry (18). In a 27-year study, 1985–2011, of stroke incidence in people under 55 years of age, they show smoking to be the most frequent risk factor (43%). However, in contrast to our study, they found an increase in the overall stroke incidence in both men and women over the time studied (18). The difference in smoking between men and women varies substantially between countries. A Chinese study of transient ischemic attack (TIA) found 41.3% of male and 4.2% of female patients to be smokers (19).

Stroke is uncommon in younger people and thus small changes in the number of events can lead to large fluctuations in incidence. However, it shows that the worrisome rapidly increasing stroke trend particularly in young women described by Rosengren et al. in 2013 has not continued (6). In contrast to our results, a recent study by Åberg et al. showed stroke incidence among young males to be increasing in Sweden (20). Their study was based on cohorts of men enrolled for the military service in 1971–1995, with the last year of their study being in 2016. Our present study lasted 2 years longer, to 2018. The most comparable group in our study (18–44 years of age), did show a higher incidence, especially for men, in 2016 but not in 2017 compared to 2005.

A study based on insurance claims data 2001-2014 from the US showed an incidence rate ratio of 0.7 for men aged 25–44 years compared with women (21). This may reflect differences between countries and also in risk factors. In the US, the prevalence of overweight was estimated to be 42% in men and women in 2017–2018 (22). While in Sweden, the prevalence of overweight was 42% in men and 28% in women in 2016 (23). The US study included patients with health care insurance which may have introduced a bias. Furthermore, misclassification may be present (24). Riksstroke is based on discharge diagnosis and as patients receive follow-up questionnaires, the risk of misclassification may be lower (25).

Previous reports have overall shown varying results regarding sex differences in fatality in stroke. A large meta-analysis from 2013 found an overall hazard risk of 1.13 for women compared with men but was unable to analyze age-adjusted differences due to limited data (26). A study from a French stroke registry showed higher mortality in women, increasing mortality with age, and no sex differences in case fatality when the results were age-adjusted (2). Our results with no difference in 90 days case fatality between the sexes are in accordance with the latter finding.

The strengths of this study are firstly the large sample size (*n* = 16,210) of the unselected cohort and high coverage of cases during the entire study period. There was also a very low number of missing values. As in all observational studies, it is difficult to establish causation between measures and outcomes, and there may be unmeasured confounding not corrected for.

The included confounders were considered the most important. In a study such as this, focusing on a younger population, some other risk factors, such as migraine with aura, the use of contraceptive pill with estrogens, pregnancy complications, obesity, and different inflammatory-related diseases such as rheumatic diseases, become interesting as many of the patients had none of the most common pre-stroke risk factors. However, to maintain a high coverage, the data collections in Riksstroke are restricted.

## Conclusion

The result of this registry-based study indicates that there are sex differences regarding the incidence of ischemic stroke in the younger age group. Both stroke incidence and many risk factors such as atrial fibrillation, diabetes, and the use of anti-hypertensives are more common in men than women. However, in younger patients, there is no significant difference in outcome between the sexes. Also, stroke incidence in the young is not decreasing as in the older age group. However, at least in Sweden, the previously reported increase in incidence seems to be reversed. Nevertheless, stroke prevention, particularly smoking, as well as increased knowledge and recognition of more uncommon risk factors must be encouraged in younger adults for us to see a decline in stroke incidence in this age group.

## Data Availability Statement

The data analyzed in this study was obtained from Riksstroke–The Swedish Stroke Register, the following licenses/restrictions apply: Due to the sensitive nature of the data, requests to access these datasets from qualified researchers trained in human subject confidentiality protocols must first be sent to Riksstroke for approval. Requests to access these datasets should be directed to Riksstroke, riksstroke@regionvasterbotten.se.

## Ethics Statement

This study was approved by the Swedish Ethical Review Authority (reference no. 2018-02777). Written informed consent for participation was not required for this study in accordance with the national legislation and the institutional requirements.

## Author Contributions

KN, ME, and MvE contributed to conception and design of the study. ME organized the database. KN performed the statistical analysis with support of ME and MvE. KN wrote the first draft of the manuscript under supervision of MvE. All authors contributed to manuscript revision, read, and approved the submitted version.

## Funding

Örebro University funded open access publication fees.

## Conflict of Interest

The authors declare that the research was conducted in the absence of any commercial or financial relationships that could be construed as a potential conflict of interest.

## Publisher's Note

All claims expressed in this article are solely those of the authors and do not necessarily represent those of their affiliated organizations, or those of the publisher, the editors and the reviewers. Any product that may be evaluated in this article, or claim that may be made by its manufacturer, is not guaranteed or endorsed by the publisher.
