# Supplementary material for: Sex Differences in Ischemic Stroke Within the Younger Age Group: A Register-Based Study
Source: Front Neurol. 2022 Feb 14;13:793181. doi: 10.3389/fneur.2022.793181 (PMC8882967; doi:10.3389/fneur.2022.793181)
Supplement: Supplementary file 1 [file Table_1.docx]

**Supplemental material Norman**

**Table 1**. Sex-specific pre-stroke risk factors subdivided in 3 age-groups

|  | Age 18-29 | | Age 30-41 | | Age 42-54 | |
| --- | --- | --- | --- | --- | --- | --- |
| **Risk factors** | Men | Women | Men | Women | Men | Women |
| Atrial fibrillation  N (%) | 7  (2.2) | 4  (1.3) | 41  (2.9) | 21  (2.0) | 558  (6.7) | 179  (3.9) |
| Diabetes  N (%) | 14  (4.3) | 10  (3.2) | 163  (11.5) | 84  (7.8) | 1493 (17.9) | 723  (15.5) |
| Hypertensive medication  N (%) | 12  (3.7) | 10  (3.2) | 207  (14.6) | 121 (11.3) | 2852 (34.3) | 1454 (31.3) |
| Smoking  N (%) | 58 (17.7) | 59  (18.4) | 328  (23.0) | 244 (22.7) | 2626 (31.3) | 1609 (34.4) |
| Previous stroke  N (%) | 17  (5.2) | 18  (5.7) | 119  (8.4) | 100  (9.3) | 993  (11.9) | 558  (12.0) |
